# Adiposity trajectories and cardiovascular disease risk in women: a population-based cohort study with a focus on menopausal status

**DOI:** 10.3389/fendo.2024.1389330

**Published:** 2024-05-24

**Authors:** Faegheh Firouzi, Fahimeh Ramezani Tehrani, Alireza Kaveh, Maryam Mousavi, Fereidoun Azizi, Samira Behboudi-Gandevani

**Affiliations:** ^1^ Tehran Medical Branch, Islamic Azad University, Tehran, Iran; ^2^ Reproductive Endocrinology Research Center, Research Institute for Endocrine Sciences, Shahid Beheshti University of Medical Sciences, Tehran, Iran; ^3^ The Foundation for Research & Education Excellence, Vestaria Hills, AL, United States; ^4^ Endocrine Research Center, Research Institute for Endocrine Sciences, Shahid Beheshti University of Medical Sciences, Tehran, Iran; ^5^ Faculty of Nursing and Health Sciences, Nord University, Bodø, Norway

**Keywords:** body mass index (BMI), body roundness index (BRI), cardiovascular disease (CVD), conicity index (CI), trajectory, waist circumference (WC), adiposity indices, tehran lipid and glucose study (TLGS)

## Abstract

**Objectives:**

A single measurement of adiposity indices could predict the incidence of cardiovascular disease (CVD); nonetheless their long-term pattern and its association with incident CVD are rarely studied. This study aimed to determine distinct trajectories of adiposity indices among participants of Tehran Lipid and Glucose Study (TLGS) and their association with incident CVD. Furthermore, this study aimed to investigate whether this association differed among individuals according to their menopausal status.

**Method:**

A total of 6840 women participated in TLGS, aged 20 years and older were included in this study; they were followed for a median of 16 years. Body mass index (BMI), waist circumference (WC), conicity index (CI) and body roundness index (BRI) were included in the analysis as adiposity indices. The cohort outcome panel of medical specialists identified the CVD outcomes. Trajectory analyses were used to identify homogeneous distinct clusters of adiposity indices trajectories. The association between the trajectory group membership and incident CVD were explored by Cox proportional hazard models, with unadjusted and adjusted model for baseline age, physical activity, smoking status, menopause and family history of CVD.

**Results:**

Three BMI trajectory groups of low, medium, and high and two trajectories for WC, BRI and CI were identified. Adjusted cox proportional hazard models revealed significant associations between the hazard of CVD experience and the high trajectory group of the BMI (HR: 2.06, 95% CI: 1.38-3.07), WC (HR: 2.71, 95% CI: 1.98-3.70), CI (HR: 1.87, 95% CI: 1.26-2.77) and BRI (HR: 1.55-95% CI: 1.12-2.15), compared to the low trajectory group. Subgroup analysis based on the menopausal status of participants showed that the HR of CVD incidences for all of trajectories adiposity indices, except BMI, was statistically significant. Adjusted cox proportional hazard models, in those women not reached menopause during study, revealed that the HR (95% CI) of CVD incidences for high trajectory of BMI, WC, CI and BRI were 2.80 (1.86-7.05); 2.09 (1.40-6.16); 1.72 (1.42-5.61), and 3.09 (1.06-9.01), respectively. These values for those were menopause at the initiation of the study were 1.40 (1.11, 2.53); 1.65 (1.04-2.75); 1.69 (1.01-2.87), and 1.61 (0.98-2.65), respectively.

**Conclusion:**

Our findings suggest that adiposity trajectories, particularly central adiposity index of CI, could precisely predict the CVD risk. Consequently, preventive strategies should be tailored accordingly.

## Introduction

The impact of obesity on cardiovascular disease (CVD) is well-documented (1, 2). Obesity contributes directly to incident cardiovascular risk factors, including dyslipidemia, type 2 diabetes, hypertension, and sleep disorders. Obesity is often associated with poor diet quality and reduced physical activity, both of which independently increase CVD risk (3). The association between adipose tissue and CVD appears to be causal, involving direct mechanisms and indirect pathways mediated through obesity-related comorbidities.

Several indicators have been developed for assessment of general, central, and visceral obesity; that predict CVD risks (4). Body Mass Index (BMI) is a widely used measure to assess general obesity; it is strongly correlated with body fat percentage using bioelectrical impedance and dual energy X-ray absorptiometry (DXA). Waist circumference (WC) is considered a superior predictor of cardiovascular disease (CVD) compared to BMI, especially among women. Several studies have shown that WC is a strong predictor of CVD and CVD-related mortality, with a higher relative risk of CVD mortality in any BMI category (5, 6). Body roundness index (BRI), and conicity index (CI) are additional indicators that have been proposed to assess obesity (7); however, the evidence on the predictive value of these indices for cardiovascular disease (CVD) risk is limited and inconsistent.

In addition to obesity, the impact of menopause on cardiovascular disease (CVD) is significant, as CVD becomes the leading cause of death in women after menopause. Several factors contributing to the increased CVD risk in menopausal women include changing hormonal milieu and endogenous sex lipids and lipoproteins; visceral obesity and revising of body fat distribution; and pro-inflammatory and pro-oxidative effects of menopause (8–10).

There are studies highlighted the importance of considering changes in cardiovascular risk factors over time and their impact on the risk of CVD (11). The Whitehall II cohort study (12), the ATTICA epidemiological study (13), the Young Finns Study (14) found that changes in BMI and waist circumference were associated with an increased risk of CVD, independent of baseline measurements. However, the long-term patterns of obesity index and their association with the incidence of CVD, considering the effect of menopause, are poorly characterized. We aimed to identify distinct trajectories of adiposity indices among participants of population-based study of Tehran Lipid and Glucose Study (TLGS) and determine their association with incident CVD and examine whether the association differed among individuals according to their menopausal status.

## Material and methods

This study was conducted according to the guidelines of the Declaration of Helsinki, and all its procedures involving human subjects were approved by the ethics committee of the Research Institute for Endocrine Sciences, Shahid Beheshti University of Medical Sciences (approval code: IR.SBMU.ENDOCRINE.REC.1402.064). Written informed consent was obtained from all subjects.

For the purpose of the present study, we used data collected in the Tehran Lipid and Glucose Study (TLGS), a prospective, population-based study aimed at assessing the prevalence and determinants of risk factors for non-communicable diseases (15). The methodology of various components of TLGS study has been previously reported (16–18).

**Figure 1:**
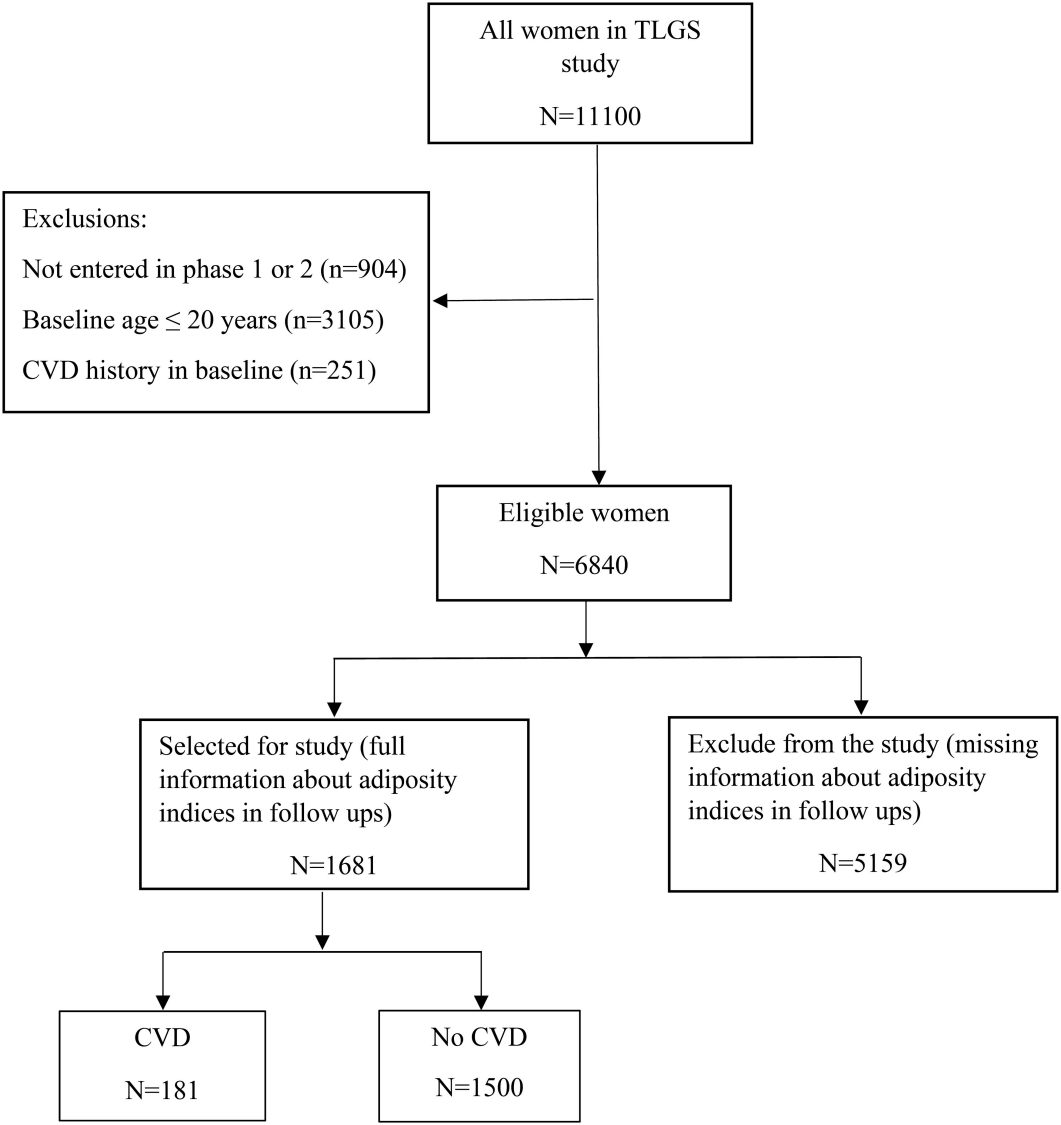
Flowchart of the study.

A total of 6840 women, >20 years old, and without history of CVD at initiation of the study, were identified. After exclusion of participants with missing information on adiposity indices or CVD status during follow-up (n = 5159), in all 1681 subjects were included in study. There were 181 events of CVD during the follow ups. All participants were present at the baseline of the study and had six follow-up visits (**Figure 1**).

Additionally, the population was stratified based on menopausal status into three subgroups of women who were menopausal before the first follow-up (n =364), women who became menopausal during the follow-up period (n = 749), and non-menopausal women throughout the follow-ups (n = 568).

In the framework of TLGS, the demographic, clinical, biochemical, anthropometric, and lifestyle data of the participants were collected at intervals of three years, starting from 1999-2001 (phase I). Further prospective follow-ups were held from 2002-2005 (phase II), 2006-2008 (phase III), 2009-2011 (phase IV), 2012-2015 (phase V), 2016-2019 (phase VI) and 2020-2023 (phase VII). In order to conduct biochemical assessments, blood samples were collected after a 12- to 14 hours overnight fast (between 7:00 and 9:00 a.m.).

All measurements were performed at baseline and at each follow-up according the standard protocol of TLGS, which has been addressed elsewhere (15). While the subjects were standing barefoot against a wall with shoulders in the normal alignment, a tape stadiometer was used to measure height. Weight was measured using a digital electronic scale (Seca 707; range 0.1–150 kg, Hanover, MD, USA) while the subjects were minimally clothed and barefoot, and the obtained weight was rounded to the nearest 100 g. Waist circumference was measured using a non-flexible tape meter at the umbilical level without pressuring the body surface while the subjects were standing, at the level midway between the lower rib margin and the iliac crest with participants in standing position, the measurements were rounded to the nearest 0.1 cm. We used the following adiposity indices: BMI (body mass index), CI (cone index), WC (waist circumference) and BRI (body roundness index).

Details of the collection of CVD events data have been published elsewhere (19). In brief, participants underwent annual follow-ups conducted by trained nurses via phone calls to identify any CVD events within the past year. If any events occurred a trained physician collected the necessary data through home visits and/or by reviewing hospital records. Subsequently, the diagnosis was confirmed by the Cohort Outcome Panel of medical specialists.

### Terms definitions

Body mass index (BMI) was measured as equation weight (kg)/height (m)^2^. The conicity index (CI) and body roundness index (BRI) proposed by following formulas (20, 21):


CI= WC (m)/[0.109 X√ Bodyweight (kg)/Height (m)]

$$BRI=364.2-365.5\times 1-\surd \;\left({\left(WC/\left(2\pi \right)\right)}^{2}{\left(0.5height\right)}^{2}\right)$$

Physical activity was assessed according to a modifiable activity questionnaire (MAQ) (22, 23). Smoking status was defined as current ever smoker or non-smoker. Menopause was defined as permanent cessation of menstrual bleeding for at least 12 months (24). A family history of CVD was defined as a prior diagnosis of CVD in any first-degree women relative aged < 65 years or a men relative aged < 55 years old. CVD is defined as the presence of CHD as defined priorly, or stroke; or cerebrovascular death (25). Coronary heart disease (CHD) is defined as (i) definite myocardial infarction (MI); diagnosed by evolving diagnostic electrocardiography (ECG) and positive biomarkers; or, (ii) probable myocardial infarction; diagnosed by positive ECG findings plus cardiac symptoms and signs plus missing biomarkers, or Positive ECG findings plus equivocal biomarkers; or, (iii) unstable angina pectoris; defined as new cardiac symptoms or changing symptom patterns and positive ECG findings and normal biomarkers; or (iv) angiographic proven CHD; or (v) CHD death; including definite and possible fatal MI (25).

### Statistical analysis

The baseline characteristics of participants were described and compared according to their CVD event. To determine the normality assumption, Kolmogorov–Smirnov test was used. The mean (SD) and ANOVA tests were used for variables with normal distribution, and median (IQR) and Kruskal-Wallis tests were applied for those without having normality assumptions. We reported frequencies (%) and used the Chi-squared test or Fisher exact test for categorical variables.

Trajectory analysis was used to identify homogeneous distinct clusters of adiposity indices trajectories including BMI, CI, WC, and BRI (groups of individuals following similar progressions over time) and assign individuals to unique clusters. The optimal number of clusters that provide a “good” partition (a partition where clusters are firstly compact and secondly well separated from each other) was selected by the Calinski-Harabatz criterion which indicates number of clusters with a large between-cluster variance and a small within-cluster variance (26). Three trajectory clusters were identified as optimum for BMI, while 2 trajectories were identified as optimum for waist, CI, and BRI. It should be noted that we included only women completed all seven follow-ups of the TLGS in order to provide greater accuracy and less bias in terms of the obtained trajectories (27–29).

The association between the trajectory group membership and incident CVD were explored by Cox proportional hazard models, with unadjusted model (Model 1) and adjusted model for baseline age, physical activity, smoking status, and family history of CVD (Model 2). Considering the high correlation between the extracted trajectories for the studied adiposity indices and their corresponding continuous adiposity variables, concurrent adjustment of both variables would likely introduce multicollinearity, thereby impeding the ability to discern the significant effects of variable 1. Additionally, the removal of one of the highly correlated variables can aid in mitigating variance inflation, thereby enhancing the model’s stability and interpretability. In accordance with the study’s objectives, we did not adjust the adiposity variables, thereby enabling the examination of the trajectories’ impact on CVD risk independently (30). All statistical analysis was performed using R statistical software (version 3.4.3) packages ‘survival’ and ‘kml’. The P-values less than 0.05 were considered statistically significant.

## Results

**Figure 1** presents the study flowchart for the present study. Considering the eligibility criteria, a total number of 1681 women were participated in this study and followed up with a median of 16 (IQR: 15-17) years. There was no statistically significant difference on baseline characteristics of those participants of TLGS that selected for the current study (n=1681) with those not selected (n=5159) (**Table 1**). Baseline characteristics of participants based on eligibility criteria at the initiation of study and the CVD experience at the end of the study are presented in **Table 1**. The effect of baseline adiposity indices on incident CVD are presented in **Table 2**.

**Table 1:** Baseline characteristics of the participants based on selected or not selected and CVD event.

Variable		Eligible women		P value	CVD incidence at follow-up		P value
		Not Selected N=5159	Selected N=1681		No n=1500	Yes n=181	
Age (years), Median (IQR)		38 (28-51)	39 (41-49)	0.058	38 (30-47)	50 (42.5-56)	<0.001
BMI (kg/m^2^), Median (IQR)		27.05 (23.82-30.80)	27.34 (24.23-30.42)	0.376	27.07 (24.08-30.11)	29.15 (26.62-32.80)	<0.001
WC (cm), Median (IQR)		87 (78-97)	87 (79-95)	0.081	86 (78-94)	94 (86-101)	<0.001
BRI, Median (IQR)		4.55 (3.24-6.03)	4.49 (3.36-5.67)	0.056	4.29 (3.24)	5.47 (4.39-6.69)	<0.001
CI, Median (IQR)		1.21 (1.14-1.30)	1.22 (1.14-1.28)	0.061	1.20 (1.13-1.28)	1.27 (1.21-1.33)	<0.001
Physical activity, N (%)	Low	3714 (71.9)	1204 (71.6)	0.890	1073 (71.5)	131 (72.4)	0.812
	High	1445 (28.0)	477 (28.4)		427 (28.5)	50 (27.6)	
Family history of CVD, N (%)	No	3223 (83.2)	1403 (83.5)	0.835	1261 (84.2)	138 (76.7)	0.010
	Yes	651 (16.8)	278 (16.5)		236 (15.8)	42 (23.3)	
Smoking history (Yes), N (%)	No	5004 (96.9)	1624 (96.6)	0.791	1450 (96.7)	173 (95.6)	0.419
	Yes	155 (3.1)	57 (3.4)		49 (3.3)	8 (4.4)	
Menopausal age	Not menopause over follow up	1754 (34.1)	568 (33.8)	0.864	553 (36.9)	15 (8.3)	<0.001
	Menopause over follow up	2244 (43.5)	749 (44.6)		669 (44.6)	80 (44.2)	
	Menopause at baseline	1161 (22.4)	364 (21.7)		278 (18.5)	86 (47.5)	

Data are Median (IQR), or N (%).

Rates are calculated after removing missing values.

BMI, Body Mass Index; WC, Waist circumference; CI, conicity index (CI); BRI, Body roundness index; CVD, cardiovascular disease.

**Table 2:** HRs and 95% confidence intervals of for incidence CVD according to the baseline adiposity indices.

Adiposity Indices	Total women		Menopausal women before study		Menopaused during study		Non-menopausal women over study	
	Model 1	Model 2	Model 3	Model 4	Model 5	Model 6	Model 7	Model 8
	HR (95% CI) P-value	HR (95% CI) P-value	HR (95% CI) P-value	HR (95% CI) P-value	HR (95% CI) P-value	HR (95% CI) P-value	HR (95% CI) P-value	HR (95% CI) P-value
BMI	1.09 (1.06,1.12) P<0.001	1.05 (1.02,1.08) 0.001	1.05 (1.01,1.08) 0.038	1.05 (1.01,1.09) 0.047	1.08 (1.03,1.13) <0.001	1.05 (1.00,1.11) 0.040	1.09 (1.00,1.20) 0.048	1.08 (0.97,1.19) 0.148
WC	1.04 (1.03,1.06) P<0.001	1.02 (1.01,1.03) 0.001	1.02 (1.00,1.04) 0.038	1.02 (1.00,1.04) 0.044	1.03 (1.01,1.05) <0.001	1.01 (0.99,1.03) 0.074	1.05 (1.01,1.10) 0.008	1.05 (1.01,1.10) 0.025
CI	2.44 (1.94,3.06) P<0.001	1.39 (1.04,1.85) 0.024	1.42 (1.11,2.12) 0.036	1.25 (1.08,1.92) 0.035	1.79 (1.9,2.67) 0.004	1.30 (0.94,2.01) 0.113	2.35 (1.11,4.98) 0.025	2.32 (1.10,4.36) 0.045
BRI	1.37 (1.27,1.48) P<0.001	1.16 (1.06,1.27) 0.001	1.14 (1.01,1.30) 0.037	1.12 (1.05,1.29) 0.036	1.26 (1.11,1.42) <0.001	1.13 (1.03,1.30) 0.045	1.43 (1.07,1.91) 0.015	1.36 (1.00,1.86) 0.047

Models 1, 3, 5, and 7 are crude models. Model 2 is adjusted for Baseline age, Physical activity, Smoking history, Menopausal status, parity and Family history of CVD. Models 4, 6 and 8 are adjusted for Physical activity, Smoking history, parity and Family history of CVD. Ref, reference category.

During the follow ups, 181 participants (10.8%) experienced CVD. There were statistically significant differences in age, BMI, WC, BRI, CI, Family history of CVD, and menopausal age between those who experienced CVD and those ones did not (P-value < 0.001). Physical activity and smoking status showed no statistically difference in CVD incidence categories (**Table 1**).

**Figure 2:**
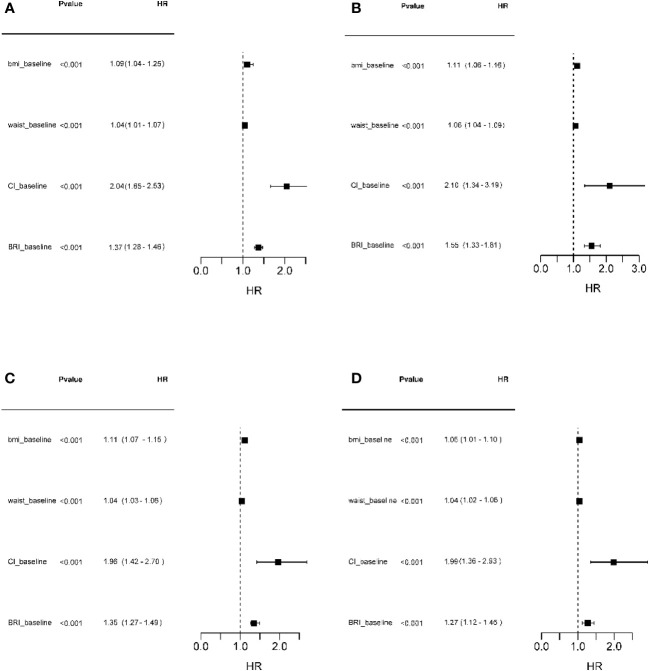
Forest plot of HRs (95% CIs) for adiposity indices (BMI, WC, CI, and BRI) at baseline for incidence CVD in total participants and subgroups according to the menopausal status. **(A)** Total participants; **(B)** Not reached menopause during follow ups; **(C)** Reached menopause during follow ups; **(D)** Menopause at baseline. BMI, Body Mass Index; WC, Waist circumference; CI, conicity index (CI); BRI, Body roundness index; CVD, cardiovascular disease.

**Figure 2** presents the forest plot of HRs (95% CIs) for baseline adiposity indices (BMI, WC, CI, and BRI) for incidence CVD in total and according to their menopausal status. As shown, with each unite increase in CI, the HR for incidence CVD among all participants (regardless of menopausal status) is 2.04 (95% CI: 1.65-2.54); it is 2.10 (95% CI: 1.34-3.19); 1.96 (95% CI: 1.42-2.70) and 1.99 (95% CI: 1.36-2.93) for non-menopausal, menopausal during follow up, and menopausal at baseline, respectively (**Figure 2**).

**Figure 3:**
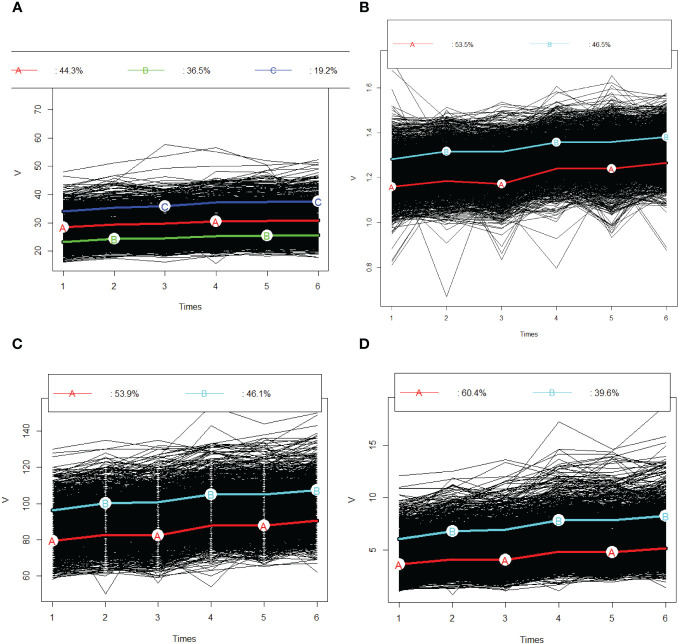
Predicted trajectories of BMI, WC, CI and BRI during follow-ups. **(A)** BMI; **(B)** CI; **(C)**; WC and **(D)** BRI. The trajectories are shown in solid line and capital letters. The proportions in each trajectory are shown above the graphs. BMI, Body Mass Index; WC, Waist circumference; CI, conicity index (CI); BRI, Body roundness index; CVD, cardiovascular disease.

**Figure 3** shows the predicted trajectories of BMI, WC, CI, and BRI during follow-ups. Trajectory models revealed three trajectories for BMI [low (36.5%, n=614), medium (44.2%, n=745), and high (19%, n=322)], two trajectories for WC [low (48.8%, n=787), and high (53.2%, n=894)], two trajectories for CI [low (53.5%, n=899), and high (46.5%, n=782)], and two trajectories for BRI [low (39.6%, n=666), and high (60.4%, n=1015)] as the best fitting of latent class growth mixture model (**Figure 3**).

**Table 2** provides definitions of the trajectories of various adiposity indices over follow ups. At the first visit, individuals in the 1st trajectory had a mean (SD) BMI of 23.14 (2.51) kg/m², which increased to 25.54 (2.43) kg/m^2^ by the last visit. For those in the medium and high trajectories, these values were 28.37 (2.22) 30.89 (2.33) kg/m², and 33.96 (3.21); 37.56 (4.65) kg/m^2,^respectively (**Table 3**).

**Table 3:** HRs and 95% confidence intervals of BMI, waist, CI and BRI trajectory groups for incidence CVD in total women.

Trajectory		Total women		Menopausal women before study		Menopaused during study		Non-menopausal women over study	
		Model 1	Model 2	Model 3	Model 4	Model 5	Model 6	Model 7	Model 8
		HR (95% CI) P-value	HR (95% CI) P-value	HR (95% CI) P-value	HR (95% CI) P-value	HR (95% CI) P-value	HR (95% CI) P-value	HR (95% CI) P-value	HR (95% CI) P-value
BMI (ref: Low)	Medium	1.43 (1.09-2.08) 0.039	1.14 (1.00-1.37) 0.048	1.57 (1.10-2.24) 0.030	1.31 (1.05-2.14) 0.021	1.73 (0.91-3.28) 0.089	1.01 (0.58-1.70) 0.357	1.68 (0.57-4.25) 0.589	1.53 (0.55-2.19) 0.384
	High	2.94 (2.00-4.32) <0.001	2.06 (1.38-3.07) <0.001	2.77 (1.50-3.68) <0.001	2.32 (1.17-3.07) 0.001	2.76 (1.90-4.18) <0.001	1.40 (1.11-2.53) 0.027	3.37 (2.28-6.38) 0.022	2.80 (1.86-7.05) 0.048
WC (ref: Low)	High	2.31 (1.42-3.75) <0.001	2.71 (1.98-3.70) <0.001	2.31 (1.42-2.75) 0.001	1.65 (1.05-2.75) 0.036	2.31 (1.42-3.75) <0.001	1.65 (1.04-2.75) 0.043	2.26 (1.20-6.35) 0.041	2.09 (1.40-6.16) 0.018
CI (ref: Low)	High	3.94 (2.81-5.52) <0.001	1.87 (1.26-2.77) 0.003	1.89 (1.10-5.95) 0.044	1.72 (1.32-5.61) <0.001	2.41 (1.49-3.89) <0.001	1.69 (1.01-2.87) 0.045	1.89 (1.30-5.95) 0.035	1.72 (1.42-5.61) 0.046
BRI (ref: Low)	High	3.00 (2.21-4.06) <0.001	1.55 (1.12-2.15) 0.004	2.33 (1.48-3.68) <0.001	1.60 (1.37-2.63) 0.026	2.33 (1.48-3.68) <0.001	1.61 (0.98-2.65) 0.056	3.32 (1.18-9.33) 0.022	3.09 (1.06-9.01) 0.040

Models 1, 3, 5, and 7 are crude models. Model 2 is adjusted for baseline age, Physical activity, Smoking history, Menopausal status, parity, and Family history of CVD. Models 4, 6 and 8 are adjusted for baseline age, Physical activity, Smoking history, parity, and Family history of CVD. Ref, reference category.

Unadjusted cox proportional hazard models (model 1) revealed significant associations between the hazard of CVD experience and the high trajectory group of the BMI (HR: 2.94, 95% CI: 2.00-4.32) compared to the low trajectory group of BMI in total participant population (**Table 3**). Results remained unchanged after adjustment for age, physical activity, family history of CVD, menopause, and history of smoking (HR: 2.06, 95% CI: 1.38-3.07). Our results also revealed that there was a significant relation between hazard of CVD experience and high trajectory group of WC in compare with low trajectory group in unadjusted model (HR: 2.31, 95% CI: 1.42-3.75) and adjusted model (HR: 2.71, 95% CI: 1.98-3.70) (**Table 3**).

The relation between high trajectory groups of CI in compare with low trajectory groups with hazard of CVD incidence was significant in unadjusted (HR: 3.94, 95% CI: 2.81-5.52), and adjusted model (HR: 1.87, 95% CI: 1.26-2.77). We also found that the relation between high trajectory groups of BRI in compare with low trajectory groups with hazard of CVD incidence was significant in unadjusted (HR: 3.00, 95% CI: 2.21-4.06) and adjusted model (HR: 1.55-95% CI: 1.12-2.15) (**Table 3**).

**Table 3** presents the unadjusted and adjusted cox proportional hazard models of CVD incidences and trajectory group of adiposity indices according to the menopausal status of participants. As it shows, after adjustment for age, physical activity, family history of CVD and history of smoking, the HR of CVD incidences for all of trajectories adiposity indices, except BMI, was statistically significant. Adjusted cox proportional hazard models, in those women not reached menopause during study, revealed that the HR (95% CI) of CVD incidences for high trajectory of BMI, WC, CI and BRI were 2.80 (1.86-7.05); 2.09 (1.40-6.16); 1.72 (1.42-5.61), and 3.09 (1.06-9.01), respectively (**Table 3**). These values for those were menopause at the initiation of the study were 1.40 (1.11, 2.53); 1.65 (1.04-2.75); 1.69 (1.01-2.87), and 1.61 (0.98-2.65), respectively (**Table 3**).

## Discussion

Our study describes the longitudinal patterns of adiposity indices and their association with CVD events among Iranian women in a prospective population-based cohort study. Using longitudinal data across seven visits, we identified three distinct trajectory groups for BMI as low, medium, and high increasing, and two trajectories for CI, WC, and BRI as low and high increasing. In all trajectory groups, adiposity indices changed in unfavorable directions. As expected, the high groups had a significantly higher CVD incidence than the low group, or medium group (for BMI). After stratification for menopausal status, we found that, regardless of menopausal status, the high trajectories of BMI, CI, WC and BRI had a significantly higher CVD incidence than the low group, and it remained significant after further adjustment for age, physical activity, family history of CVD and history of smoking. The increased risk is particularly pronounced in participants who had not yet reached menopause during the study period. All baseline adiposity indices were significantly associated with increased HRs for CVD events in both the total participant group and in all subgroups stratifies by menopausal status. Among these indices, CI was associated with the highest risk of CVD incidence, with each unit increase in CI corresponding to approximately a two-fold increase in CVD incidence.

In our study, we showed that single measurement of adiposity indices at baseline, including BMI, WC, RBI and CI was strongly associated with developing CVD across the time; this risk was more pronounced among individuals with central obesity indices including CI and RBI; for each unit increase in CI, there was an approximately twofold increase in the incidence of CVD. In line with this finding, Nkwana et al. (2021) in a cross-sectional study reported that compared to other central adiposity indices such as A body Mass Index (ABSI), BRI, the CI had the strongest associations with CVD risk factors including insulin resistance, hypertension, and dyslipidemia (7).

However, most studies have only investigated adiposity indices measured at a single point or as an average value over a period, which may not completely capture the longitudinal changes in the risk of CVD events. Single measurements fail to account for the dynamic nature of adiposity and its impact on cardiovascular events over time. It is important to note that individuals identified as obese based on a single measurement may not experience uniform risk of cardiovascular disease (CVD) events, given the variability of their obesity status over time. Therefore, it is crucial to consider the long-term patterns of obesity when assessing CVD risk in individuals. The trajectory of obesity over the lifespan, particularly during critical periods such as reproductive years, menopause transition, and menopause can significantly influence these risks (31). Therefore, it is crucial to consider the long-term patterns of obesity when assessing CVD risk in individuals.

Numerous methodological advancements have emerged to examine the temporal variations of risk factors, including obesity, which has significant implications for cardiovascular disease (CVD) risk stratification. Distinct trajectory modeling techniques offer a dynamic perspective on the heterogeneity within and among individuals regarding health outcome patterns. By employing this approach, researchers can identify subpopulations at elevated risk of unfavorable outcomes, thereby surpassing the limitations inherent to single-measurement evaluations alone. Trajectory modeling provides valuable insights into the intricate interactions between obesity, critical life stages, and CVD risk (32–36).

The longitudinal design of our study allowed for repeated measurements of the variables of interest, enabling us to account for intraindividual changes over time. However, few studies have investigated the longitudinal trajectories of various obesity indices and their impact on cardiovascular disease (CVD) risk, particularly with respect to assigning individuals to unique clusters based on the timing of adiposity changes. Additionally, a significant gap exists in the literature regarding the longitudinal trajectories of novel obesity indices, such as the conicity index (CI), and their relationship to CVD risk, particularly in women based on their menopausal status.

The longitudinal design of our study allowed for repeated measurements of the variables of interest, enabling us to account for intraindividual changes over time. However, few well designed studies have investigated the longitudinal trajectories of various obesity indices and their impact on CVD risks, particularly with respect to assigning individuals to unique clusters based on the timing of adiposity changes. Furthermore, a significant gap exists in the literature regarding the longitudinal trajectories of novel obesity indices such as CI, and their relationship to CVD risk, particularly in women based on their menopausal status.

In this respect, our findings align with previous studies indicating that excess adiposity indices over time could increase the risk of CVD events (14, 37–46). We observed that individuals in the high distinct trajectory groups for BMI, CI, WC, and BRI demonstrated a significantly higher incidence of CVD events compared to those in the low trajectory group. In agreement with our findings, Wang et al. (2020) showed that WC trajectory patterns were associated with altered risk of CVD among Chinese adults (41). Fan et al. reported a positive association between BMI trajectories and incident hypertension (46). Recently, Ding et al. (2023) conducted a population-based, longitudinal study, involving 71,166 Chinese individuals with a median follow-up of 7.93 years, investigating the association of BRI and its longitudinal trajectories with cardiovascular mortality. Their study identified three BRI longitudinal trajectories of low-, moderate-, and high-stable. After adjustment for potential confounders, they found that the HRs for CVD mortality was 1.12 (95% CI: 1.05–1.18) for the moderate-stable group and 1.64 (95% CI: 1.53–1.75) for the high-stable group compared to the low-stable group (45). In another well-designed study, Wu et al. (2022) examined BRI trajectories and their associations with incident CVD events among 59,278 participants. They categorized the BRI trajectories into 4 distinct groups of low-, moderate-, moderate-high-, and high-stable. Following adjustment for potential confounders, individuals in the moderate-stable group exhibited an HR of 1.37 (95% CI: 1.19-1.58) for CVD compared to those in the low-stable group. Similarly, participants in the moderate-high-stable group demonstrated an HR of 1.64 (95% CI: 1.40-1.91), while those in the high-stable group displayed the highest HR of 2.03 (95% CI: 1.64-2.52) for CVD relative to the low-stable group. Their findings indicated a significant association between BRI trajectories and the risk of CVD (43).

Nevertheless, it is imperative to acknowledge that the observed number of trajectories in the existing studies varies from those identified in our present investigation. This disparity may stem from differences in sample size, the inherent heterogeneity within the study population, the duration of observation, the complexity of the employed statistical models, and the specific definition of trajectories. Furthermore, the assignment of individuals to distinct clusters or the consideration of specific time periods during which changes in adiposity occur, along with the adjustment for various covariates, are factors that may contribute to the divergence in trajectory patterns across studies.

Moreover, our study provides novel evidence that, despite the significant association between high trajectories of adiposity indices and increased cardiovascular disease (CVD) risk being consistent across menopausal status, a more pronounced increase in risk is observed among participants who had not yet reached menopause during the study period. It appears that the relationship between obesity and cardiovascular disease (CVD) risk is complex and multifactorial, and may vary depending on a range of factors, including menopausal status, duration of obesity, and the specific CVD outcome being considered; the trajectories of obesity indices and cardiovascular disease (CVD) may change by menopause (47). While some studies have suggested that the increased risk of CVD associated with obesity may be more pronounced among menopausal women, other studies have reported conflicting findings. For example, a recent population-based study found that obesity was associated with a reduced risk of mortality in postmenopausal women, but not in premenopausal women (48). It is possible that the observed paradoxical relationship between menopausal status and the impact of obesity on CVD risk may be due to differences in the underlying mechanisms driving CVD risk in pre- and postmenopausal women. For example, premenopausal women may have a higher baseline level of estrogen, which has been shown to have a protective effect against CVD. Therefore, the loss of this protective effect during menopause may contribute to the observed increase in CVD risk among postmenopausal women. Moreover, menopause is associated with increased abdominal and visceral obesity, which increases cardiometabolic risk and mortality. Nonetheless, we posited that subjects who underwent menopause prior to the commencement of the study or attained menopause during the follow-up period had likely encountered the detrimental consequences pertaining to both menopause and obesity on CVD risk. Contrarily, our observations indicated that the risk of CVD was markedly amplified amongst female young premenopausal adults who had not yet attained menopause, thus having evaded the adversarial impacts of menopause on CVD, whilst simultaneously encountering progressive weight gain and central obesity. As a result, female young adults exhibiting such trajectories were more prone to developing CVD events.

The strengths of this study should be addressed. The long-term prospective design, which involved a large, population-based cohort, rigorous follow-up procedures, the utilization of accurate and valid data, along with adjustment for a wide range of potential confounders are the main strengths of this study. Moreover, our study also employed multiple measurements collected over time across seven visits, rather than relying on a single measurement at a specific life stage, which allowed us to utilize the trajectory approach, enhancing accuracy and capturing dynamic changes in adiposity over the study period. No other studies investigated the influence of CI trajectories on CVD over an extended follow-up period involving seven subsequent examinations, as was done in our study. Additionally, restricting our population to only female participants, as an important source of heterogenicity in population, enabled us to uncover nuanced trajectory patterns that manifest over extended periods. This approach also allowed us to evaluate the influence of menopause on these trajectories.

The study has some limitations as well. Primarily, our study included only women completed all seven follow-ups of the TLGS, leading to the exclusion of a considerable number of female participants from the TLGS cohort. However, using this approach helped us to provide to provide greater accuracy and less bias in terms of the obtained trajectories (27–29). Nonetheless, as we showed, there were no statistically significant difference between baseline characteristics between the study participants who were excluded and those who were included. The generalizability of our study findings to other populations may be limited due to the specific Persian heritage of the study group. In addition to adjusting for multiple covariates, residual confounding remains a potential concern. The role of life style modifiers have not been considered in present study. We did not evaluate alternative robust adiposity indices, such as dual-energy x-ray absorptiometry (DXA)-derived body composition metrics, magnetic resonance imaging (MRI)-measured adipose tissue depots, and bioelectrical impedance analysis (BIA)-estimated lean and fat masses. These indices could potentially yield complementary information about the link between obesity and CVD risk. Furthermore, the menopausal age was self-reported; however, we confirmed the reliability of the data on menopausal age by asking the women the same questions 3 years later; therefore, the effect of recall error is minimal.

In conclusion, using population-based data over a median follow-up period of 16 years, we identified multi-trajectory groups for BMI, WC, CI, and BRI groups in the Iranian women and examined associated risks of CVD events. The high increasing trajectories of BMI, WC, BRI, and CI groups were associated with higher risks of CVD incidence than the low group regardless of menopause status of women. Among these indices, CI was associated with the highest risk of CVD incidence. The increased risk was more evident among participants who had not reached menopause during the study period. Further research is needed to fully elucidate the underlying mechanisms driving these observed differences in CVD risk between pre- and postmenopausal women. Considering the unique changes in body composition and fat distribution that occur throughout the lifespan, investigating the longitudinal trajectories of the CI may provide valuable insights for developing targeted prevention and intervention strategies.

## Data availability statement

The raw data supporting the conclusions of this article will be made available by the authors, without undue reservation.

## Ethics statement

The studies involving humans were approved by the ethics committee of the Research Institute for Endocrine Sciences, Shahid Beheshti University of Medical Sciences (approval code: IR.SBMU.ENDOCRINE.REC.1402.064). Written informed consent was obtained from all subjects. The studies were conducted in accordance with the local legislation and institutional requirements. The participants provided their written informed consent to participate in this study.

## Author contributions

FF: Writing – review & editing, Writing – original draft, Data curation, Conceptualization. FRT: Writing – review & editing, Writing – original draft, Supervision, Methodology, Funding acquisition, Conceptualization. AK: Writing – review & editing, Writing – original draft, Investigation. MM: Writing – review & editing, Writing – original draft, Methodology, Formal analysis. FA: Writing – review & editing, Writing – original draft, Investigation. SB-G: Writing – review & editing, Writing – original draft, Methodology, Funding acquisition, Conceptualization.
